# Coenzyme Q10 modulates the immunity by enhancing mononuclear macrophage, NK cell activity, and regulating gut microbiota

**DOI:** 10.3389/fnut.2025.1504831

**Published:** 2025-03-17

**Authors:** Yajun Liang, Yang Han, Ling Xiao, Yupeng Su, Tongen Bao, Xia Ji, Longgang Jia, Jing Zhang

**Affiliations:** ^1^College of Pharmacy, Qilu Medical University, Zibo, China; ^2^College of Food Science and Engineering, Tianjin University of Science and Technology, Tianjin, China

**Keywords:** Coenzyme Q10, immunity, carbon clearance ability, natural killer cells, gut microbiota

## Abstract

**Introduction:**

Coenzyme Q10 (CoQ10), an important fat-soluble, bioactive molecule that predominantly found in the inner mitochondrial membrane, is widely used in functional food and health food raw materials, which has garnered considerable attention due to its potential role in immunoregulation. However, the intrinsic mechanism of CoQ10 on immunity, and the relationship to the gut microbiota have not been elucidated.

**Methods:**

Here, we conducted a series of *in vivo* experiments with the aim of comprehensively exploring the effect of CoQ10 on both cellular and humoral immune functions, and on gut microbiota communities in mice.

**Results:**

CoQ10 showed negligible impact on both mouse body weight fluctuations and tissue indices, but enhanced the mouse body immunity by elevating the carbon clearance ability and natural killer (NK) cellular viability. 16S rRNA gene sequencing revealed that administration of CoQ10 modulated the structure and composition of the gut microbiota in mice, notably by enhancing the abundance of *Lactobacillus, Limosilactobacillus*, and decreasing the abundance of *Paramuribaculum* species.

**Discussion:**

This work makes a contribution to the application of CoQ10 as an immunomodulator in the biological, pharmaceutical and health care product industries.

## 1 Introduction

Natural compounds, including dietary supplements and bioactive molecules, have emerged as promising agents for immune and gut microbiota regulation (1), glycemic control and liver function (2), oxidative stress, type II diabetes, cardiovascular diseases, and other chronic diseases (3). Due to the side effects and toxicity, the natural compound shows the potential for better alternative to conventional medicines. As a natural compound, Coenzyme Q10 (CoQ10), a fat-soluble, bioactive compound primarily residing within the inner mitochondrial membrane, serves as a pivotal electron transporter in the electron transport chain, thereby sustaining the vital proton gradient across this membrane (4). Moreover, CoQ10 is also a potent lipophilic antioxidant (5). The fat-soluble nature of CoQ10 enables it to safeguard cell membranes, lipoproteins, and DNA from the detrimental effects of free radical-mediated oxidative stress (6, 7). Given that phagocytes, comprising macrophages, neutrophils, are primarily responsible for eliminating invading pathogens by generating free radicals, CoQ10’s antioxidant capabilities offer a degree of protection against the self-destructive effects of these reactive species (8). More important, emerging studies have shown that CoQ10 has great potential for improving immunity (9–11).

The intestines of mammals are colonized by a large number of microorganisms, which are collectively known as the intestinal flora. Advancements in high-throughput sequencing technology have facilitated the identification and in-depth functional analysis of microbial communities. Most of the gut bacteria in the body belong to *Firmicutes*, *Bacteroidetes*, *Proteobacteria*, or *Actinomycetes* (12). The gut microbiota is diverse, and there are great differences between individuals depending on feeding patterns, lifestyles, medications, and host genes (1, 13). The gut microbiota of the same individual can also change at different ages, health conditions, or dietary habits (14). The long-term co-evolutionary process between the intestinal flora and its host has fostered a mutual reliance, enabling the microbiota to maintain host homeostasis through intricate regulation of nutritional status, immune development, metabolism, and protection against pathogens (15). The intestinal flora exerts a pivotal role in shaping and modulating the immune system, underscoring its fundamental importance in maintaining immunological balance. Immune development in germ-free animals is impaired, as evidenced by immature gut-associated lymphoid tissue, decreased intestinal lymphocyte numbers, and decreased levels of antimicrobial peptides and immunoglobulin IgA, all of which are reversed when colonized by commensal bacteria (16).

Findings reinforce the indispensable part that gut microbiota plays in fostering the development and maturation of the host’s immune system. This modulation occurs via the production of an array of immunomodulatory and anti-inflammatory molecules, notably short-chain fatty acids (SCFAs), indoles and their derivatives, along with secondary bile acids. These molecules intricately orchestrate immune cell responses, spanning T cells, B cells, dendritic cells, and macrophages (17–21). For instance, SCFAs facilitate the generation of IL-10 in Th1 cells via G-protein-coupled receptor 43 (GPR43) engagement (17), stimulate IL-22 production in CD4+ T cells and innate lymphocytes through G-protein-coupled receptor 41 (GPR41), and concurrently inhibit histone deacetylases (18). Secondary bile acids interact with takeda G-protein-coupled receptor 5 (TGR5), resulting in reduced inflammasome activation mediated by nucleotide-binding oligomerization domain-like receptor protein 3 (NLRP3), thus inhibiting NF-κB signaling to downregulate macrophage proinflammatory cytokine production, and by nuclear receptors interacting with farnesoid X receptor (FXR) leads to the inhibition of co-repressor protein-mediated chromatin modification, which in turn inhibits the expression of NF-κB-dependent inflammatory mediators in macrophages (19). *Bacteroides fragilis* is able to induce the development of Th1 cells through its polysaccharide A, which also promotes immune tolerance by inhibiting Th17 differentiation and enhancing Treg cell activity through interacting with T cells through Toll-like receptors 2 (TLR2) (20). *Lactobacillus* nucleocytosis exhibits a complex interplay with the tumor microenvironment, modulating the infiltration patterns of tumor-infiltrating lymphocytes (TILs) and inhibiting the cytotoxic functions of NK cells and TILs towards cancer cells. Notably, this microorganism may contribute to the etiology of colorectal cancer by fostering an environment conducive to its development (22). Gut microbiota can also influence the infiltration of NK cells and T cells in the tumor microenvironment, thereby modulating the activity of these immune cells and consequently affecting the development of colorectal cancer (23).

Thus, gut microbiota is pivotal for immune system establishment and homeostasis, with dysbiosis implicated in immune-mediated disorders. Imbalances between the microbiota and immune system can precipitate diverse pathologies, notably inflammatory bowel disease (IBD) and colorectal cancer (21, 24). While the immunoregulatory and microbiota-modulating properties of CoQ10 remain under-explored, our study endeavors to fill this knowledge gap. By conducting comprehensive physiological, biochemical, and 16S rRNA gene sequencing experiments in mice, we aimed to elucidate the impact of CoQ10 on immune function and gut microbiota. The findings from this investigation offer vital insights and theoretical underpinnings for the utilization of CoQ10 as a food additive with potential implications in nutrition and health.

## 2 Materials and methods

### 2.1 *In vivo* animal experiments

Forty specific pathogen free (SPF) healthy Kunming mice (half male and half female, aged about 2 month), weighing 18 to 22 g, were provided by Pengyue laboratory animal Co., LTD (Jinan, China). In this study, different doses of CoQ10 were orally administered to the mice, tailored according to their individual body weights (BW). Specifically, according to the method for the assessment of enhancing immune function in the Technical Standards for Testing and Assessment of Health Food of China, the mice were randomly divided into four groups (10 mice in each group): control group (Ctrl), 0.05 g/kg BW CoQ10 group (LCoQ10), 0.10 g/kg BW middle-dose CoQ10 group (MCoQ10), and 0.30 g/kg BW high-dose CoQ10 group (HCoQ10). The experimental mice were housed in a rigorously controlled environment, with temperature maintained within a range of 20 to 25°C and relative humidity adjusted to an optimal level of 40 to 70%, ensuring consistent conditions throughout the study. The mice were exposed to a standardized 12-h light–dark cycle and provided with continuous access to food, thereby safeguarding their welfare. The conduct of animal experiments adhered strictly to the guidelines outlined in the National Institutes of Health (NIH) Guide for the Care and Use of Laboratory Animals, and all protocols were thoroughly reviewed and approved by the Institutional Animal Care and Use Committee at the Zibo Food and Drug Inspection Institute, under the approval number SYXK (Lu) 2013 0004. The animals were administered the AIN93M diet, sourced from Beijing Keao Xieli Feed Co., LTD (Beijing, China), which carries a valid production license number: scxk (Beijing) 2014–0010.

After a 7-day acclimatization period for the mice in their new environment, different concentrations of CoQ10 were orally administrated for 30 days. The experimental control group underwent administration of an equivalent dose of 0.9% sterile saline via gavage, mirroring the procedure applied to the treatment groups. Prior to and subsequent to the 30-day intervention period, the body weights of these mice were meticulously tracked to assess any potential changes. Following completion of the treatment regimen, the mice were humanely euthanized via cervical dislocation, in adherence to ethical guidelines. Subsequently, key biological samples including the thymus, spleen, serum, and feces were meticulously harvested for subsequent analysis and testing.

### 2.2 Visceral index calculation

The thymus and spleen tissues were drained with filter paper and weighed. Then the visceral indexes were calculated using thymus or spleen tissues’ weight divided to body weight.

### 2.3 Lymphocyte transformation assay

The spleen was meticulously placed within a sterile dish, immersed in an appropriate volume of Hank’s balanced salt solution (HBSS), and subsequently subjected to gentle disruption using forceps to achieve a homogenized single-cell suspension. This suspension underwent refinement through a 200-mesh filter. The cells were washed twice with HBSS and followed by centrifuging at 1000 rpm for 10 min. Following two rounds of centrifugation (1,000 rpm, 10 min), the cells were washed thoroughly with HBSS and then resuspended in 1 mL of complete culture medium. The viability and concentration of the cells were ascertained via trypan blue exclusion assay, and the final cell density was adjusted to 3 × 10^6^ cells/mL. Subsequently, aliquots of the splenocyte suspension were dispensed into two wells of a 24-well culture plate, with 1 mL per well. To assess lymphocyte proliferation, 75 μL of concanavalin A (ConA) solution was introduced into one well, serving as the stimulus, while the adjacent well received no such addition as the control. Both sets were incubated under optimal conditions (5% CO_2_, 37°C) for 72 h using a carbon dioxide cell incubator (HHCP-7, Boxun, Shanghai, China). Approaching the end of the incubation period, 0.7 mL of the supernatant was aspirated carefully from each well, replaced with an equal volume of Roswell Park Memorial Institute (RPMI) 1,640 medium devoid of fetal bovine serum, and supplemented with 50 μL of methylthiazolyldiphenyl-tetrazolium bromide [MTT, purchased from Sigma-Aldrich Corporation (St. Louis, MO, United States)] per well. The cultures were then further incubated for another 4 h. Upon completion, the purple formazan crystals formed were solubilized by the addition of 1 mL of acidic isopropanol to each well, followed by thorough mixing. The extent of proliferation was quantitatively assessed by measuring the OD of the solubilized product using a spectrophotometer (Varioskan LUX, Thermo Fisher Scientific, MA, United States), at a wavelength of 570 nm. To calculate the proliferation effect attributed solely to ConA stimulation, the OD value obtained from the ConA-treated well was subtracted from that of the untreated control well. This adjusted OD value served as an indicator of the lymphocytes’ proliferative capacity in response to the mitogen.

### 2.4 The delayed-type hypersensitivity (DTH) response assay

The abdominal skin of each mouse was depilated with barium sulfide, ranging from about 3 cm by 3 cm in size. Subsequently, 50 μL of dinitro-fluorobenzene (DNFB) was uniformly applied to this pre-treated skin area to initiate sensitization. Following a period of 5 days, the sensitization process was reinforced by administering an additional 10 μL of DNFB, which was carefully and evenly distributed onto both surfaces of the right ear of each mouse. Mice were euthanized 24 h subsequent to the induction of cervical dislocation, whereupon the auricular structures, specifically the left and right ear pinnae, were carefully excised. An 8 mm diameter earpiece was precisely excised utilizing a punch tool, with the subsequent discrepancy in weights between the left and right ears serving as a quantitative metric for assessing the degree of DTH. This methodology renders a test result as positive when the statistical significance of the weight variation within the experimental group markedly surpasses that observed in the control cohort, thereby providing a robust indication of the presence of DTH.

### 2.5 Hemolytic antibody plaques formation test

The defibrinated sheep blood was procured and subjected to a rigorous washing procedure using sterile normal saline solution, followed by centrifugation at 2,000 rpm for 10 min, repeated three times. Subsequently, the overconcentrated sheep red blood cells (SRBC) were diluted to a 2% (v/v) cell suspension using saline. After administering 0.2 mL of the suspension via intraperitoneal injection, each mouse underwent the immunization process. Five days subsequent to the SRBC immunization, the mice were humanely euthanized through cervical dislocation, at which point their spleens were meticulously extracted for the preparation of a splenic cell suspension. After heating and dissolving 10 g/L of agarose, it was maintained at a constant temperature in a 50°C water bath. The aforementioned solution was subsequently combined with an equivalent volume of 2 × HBSS. The mixture was dispensed into small test tubes, with each containing 0.5 mL. To each tube, 50 μL of SRBC (prepared at 10% v/v with SA (Sulfosalicylic acid) buffer) and 25 μL splenocyte suspension were added, and this mixture was promptly agitated to ensure homogeneity and then delicately dispensed onto a pre-prepared slide, which had been coated with a uniform, thin layer of agarose. By carefully manipulating the dispensed mixture, parallel strips were formed across the slide. Once the agarose solidified, the sample was secured in place, and the slide was positioned horizon-tally within a slide rack for further processing. Subsequently, the slide was transferred to a carbon dioxide-enriched incubator, where it underwent incubation for a period of 1.5 h. Then, the wave rack’s grooves were supplemented with a 1:8 dilution of complement in SA buffer. Following an additional incubation of 1.5 h, the enumeration of hemolytic plaques was meticulously performed. A positive test outcome was designated if the count of plaques observed in the test sample cohort significantly surpassed that recorded in the control group.

### 2.6 Serum hemolysin test

After the defibrinated sheep blood was meticulously gathered, it underwent rigorous washing procedures with normal saline, which was repeated three times to ensure thorough-ness. Subsequently, the concentrated SRBCs were diluted with saline to achieve a 2% (v/v) cell suspension, which was administered to each mouse via intraperitoneal injection in a volume of 0.2 mL for immunization purposes. Five days post-immunization, the eyeball was surgically extracted, and the ensuing blood was collected in a designated centrifuge tube. The tube was allowed to rest for approximately 1 h, facilitating blood coagulation. Following coagulation, the clotted blood was meticulously dislodged from the tube’s walls, and the serum was gently aspirated for subsequent analysis. To guarantee complete segregation, the serum underwent further centrifugation at 2,000 rpm for a duration of 10 min. The collected serum was then diluted with saline to various concentrations. These diluted serum samples were dispensed into a micro-hemagglutination experimental plate, with 100 μL in each well. To each well, 100 μL of 0.5% (v/v) SRBC suspension was introduced, followed by a thorough mixing procedure, and subsequently transferred onto a pre-moistened flat plate. The plate was then securely capped and incubated in a 37°C incubator for 3 h, during which time the extent of hemocytoplasm aggregation was closely monitored and observed.

The degree of serum agglutination is typically graded on a scale of 0 to IV, with each grade defined by specific criteria. Grade 0 indicates that all red blood cells settle and concentrate at the bottom of the well, forming a dense dot surrounded by clear flu-id. Grade I is characterized by red blood cells mostly concentrated in dot-shaped clusters at the bottom, with a few agglutinated cells surrounding them. In Grade II, a discernible thin layer of aggregated red blood cells becomes visible at the base, forming a distinct sediment, with a loosely packed red dot discernible in the center. In Grade III, the agglomerated cells exhibit a uniform distribution, forming a thin and even layer across the designated area, with a faintly visible small red dot in the middle. Finally, Grade IV denotes a distinctive pattern where the agglomerated red blood cells exhibit a uniform dispersion, forming a thin, even layer at the base. This configuration may concomitantly present with a visibly curled clot, indicative of a specific aggregation state.

The serum hemolysin was determined using anti-SRBC value calculated using the following formulas: Anti-SRBC=S_1_ + 2S_2_ + 3S_3_…nS_n_. In which, the S means the degree of serum agglutination; 1, 2, 3…n means the double dilution index.

### 2.7 Carbon clearance test

Inject ink, diluted with normal saline in a precise 1:4 ratio, via the tail vein of the mouse, with the dosage tailored to its individual body weight, and immediately begin timing upon injection. At 2nd and 10th min after the injection, 20 μL blood was collected from the medial canthal plexus and promptly transferred to 2 mL of a 0.1% Na_2_CO_3_ solution. Measure the OD value at wavelength of 600 nm using the 754 N ultraviolet spectrophotometer (Jingke, Shanghai, China), utilizing the Na_2_CO_3_ solution as the blank control. Subsequently, euthanize the mice and retrieve the liver and spleen. Utilize filter paper to meticulously blot any residual blood stains from the surfaces of these organs, and subsequently proceed to weigh each organ individually. The phagocytosis index A can be calculated using the following formulas:


$$K=\frac{\mathrm{lgOD}1–\mathrm{lgOD}2}{t2-t1}$$

$$\mathrm{Phagocytosis index}\;A=\frac{\mathrm{Body weight}}{\mathrm{Liver weight}+\mathrm{Spleen weight}}\times \sqrt[{3}]{K}$$

A positive test outcome was determined when the phagocytosis index, as measured in the test sample group, exhibited a statistically significant elevation compared to that observed in the control group.

### 2.8 Macrophage phagocytosis of chicken erythrocytes

Inject 1 mL of 20% chicken erythrocyte suspension into the abdominal cavity of each mouse. After 30 min, the mouse was humanely euthanized via cervical dislocation and positioned supine. The abdominal wall skin was subsequently bisected, and 2 mL of normal saline was administered into the abdominal cavity. The mouse was then rotated on its board for 1 min to facilitate the lavage process. Subsequently, 1 mL of peritoneal lavage fluid was aspirated and deposited onto a slide, which was then placed within an enamel container lined with moistened gauze to maintain humidity. This slide was incubated at 37°C for 30 min to optimize cell visualization. Upon completion of incubation, the slide was rinsed thoroughly with normal saline, air-dried, and fixed using an acetone-methanol mixture (1:1 ratio) to preserve cellular morphology. For staining, the slide was immersed in a 4% Giemsa-phosphate buffer solution for 3 min, followed by rinsing and drying with distilled water. Macrophages were enumerated under an oil immersion microscope (BX51, Olympus, Japan), with a standardized count of 100 macro-phages per slide. The phagocytic percentage and phagocytic index B were derived using the prescribed formulas:


$$\begin{array}{l} \mathrm{Phagocytic}\;\\ \mathrm{percentage}\;\left(\%\right)=\frac{\begin{array}{l} \mathrm{Macrophages that phagocytized}\\ \;\mathrm{red}\;\mathrm{blood cells} \end{array}}{\begin{array}{l} \mathrm{The counted number of}\;\\ \mathrm{macrophages} \end{array}}\times 100\% \end{array}$$

$$\mathrm{Phagocytic index}\;B=\frac{\begin{array}{l} \mathrm{Total number of engulfed}\;\\ \mathrm{chicken}\;\mathrm{red}\;\mathrm{blood cells}\; \end{array}}{\begin{array}{l} \mathrm{The counted number}\;\\ \mathrm{of macrophages} \end{array}}\times 100\%$$

The phagocytic capacity of mouse macrophages is expressed as phagocytosis percentage and phagocytosis index. The positivity of the test was ascertained when a statistically significant elevation was observed in either the percentage of phagocytosis or the phagocytosis index within the test sample group, compared to the corresponding values in the control group.

### 2.9 Assessing the functional potency of NK cells in mice using lactate dehydrogenase (LDH) method

Prior to the commencement of the experiment, YAC-1 cells underwent a 24-h cultivation period, followed by thorough washing with HBSS on three consecutive occasions. The cell density was subsequently adjusted to 4 × 10^5^ cells/mL in RPMI 1640 supplemented with a comprehensive culture medium. The mice were humanely euthanized via cervical dislocation, and their spleens were aseptically extracted for the preparation of a splenic cell suspension. This suspension was subjected to three rounds of washing with HBSS, each followed by centrifugation at 1000 rpm for 10 min. The pelleted cells were resuspended in RPMI 1640 culture medium fortified with 10% fetal calf serum, and the viability of the cells was assessed through trypan blue exclusion, and the cell concentration was adjusted to 2 × 10^7^ cells/mL. Subsequently, 100 μL aliquots of both target and effector cells (at an effector-to-target ratio of 50:1) were dispensed into the wells of a U-bottomed 96-well culture plate. For the determination of spontaneous cell death, 100 μL of target cells (ODtarget) and culture medium (OD0) were dispensed into designated wells, whereas for maximal lysis assessment (OD-max), 100 μL of target cells were mixed with 1% Nonidet P-40 (NP40). Each experiment was conducted in triplicate, and the plates were incubated in a humidified atmosphere containing 5% CO_2_ at 37°C for 4 h. Post-incubation, the plates were centrifuged at 1500 rpm for 5 min, and 100 μL of supernatant from each well was transferred to a flat-bottomed 96-well plate. Subsequently, 100 μL of LDH substrate solution was added to each well, and the reaction mixture was allowed to incubate for 10 min. To terminate the reaction, 30 μL of 1 M hydrochloric acid was added to each well, and the absorbance was measured at 490 nm using a microplate spectrophotometer. The cytotoxic activity of NK cells was quantified employing the following mathematical formula:


$$NK\;\mathrm{cell activity}\;\left(\%\right)=\frac{\mathrm{ODtarget}-OD0}{\mathrm{ODmax}-OD0}\times 100\%$$

### 2.10 Gut microbiota analysis

To investigate the effects of CoQ10 on the constitution and structure of gut microbiota, we embarked on a comprehensive analysis utilizing 16S rRNA gene sequencing (25, 26). Each sample was sequenced in a single run on the Illumina MiSeq platform, targeting the V3-V4 hypervariable regions of the 16S rRNA gene. In essence, fresh feces samples (3 in each group) from mice were procured at the conclusion of the animal studies, and subsequently, we proceeded with the extraction and quantification of the total genomic DNA content present within these meticulously selected samples. The 16S rRNA gene, specifically its highly variable V3-V4 region, underwent amplification utilizing the total DNA extracted as the foundational template. Specifically, the forward and re-verse primers utilized were 5’-CCTACGGGNGGCWGCAG-3′ and 5’-GACTACHVGGGTATCTAATCC-3′, respectively. The intricate process of high-throughput sequencing, followed by its meticulous analysis, was skillfully executed by BGI Co., Ltd. (Shenzhen, China). All samples were processed and sequenced individually to ensure the independence and accuracy of the results.

To categorize the reads, Operational Taxonomic Units (OTUs) were employed, setting a threshold of 97% sequence identity. To assess the intricacies of bacterial abundance fluctuations and gut microbiota diversity patterns, we employed a comprehensive approach encompassing *α* diversity indices, *β* diversity metrics, and Principal Co-ordinate Analysis (PCoA), offering a multifaceted view of microbial ecology. Additionally, we utilized Linear Discriminant Analysis Effect Size (LEfSe) as a powerful tool to discern and dissect the key bacterial taxa that distinguished the experimental groups, thereby facilitating a deeper understanding of the intergroup microbial variations.

### 2.11 Statistical analysis

All data were presented as (Means ± SD). Statistical difference between the control and CoQ10 groups were analyzed by Student’s *t*-test using GraphPad Prism 8.0.1 software.

## 3 Results and discussion

### 3.1 CoQ10 showed negligible impact on the mouse body weight and spleen or thymus index

**Figure 1:**
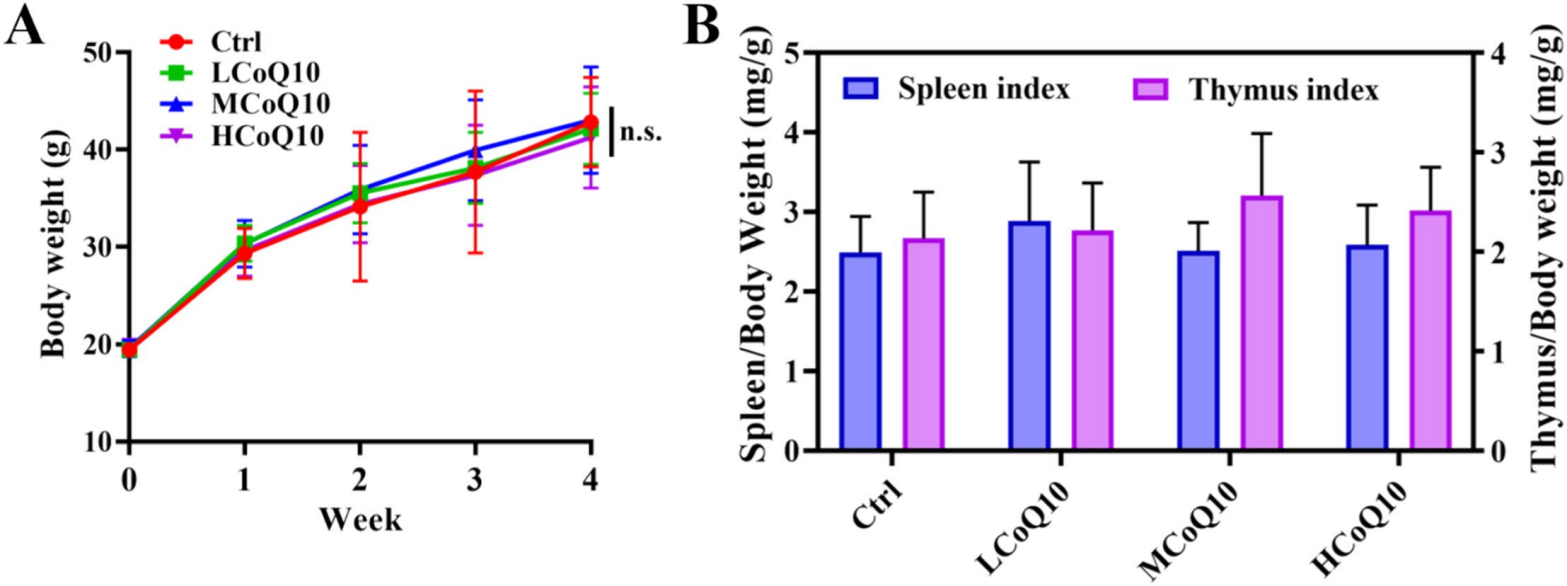
Effect of Coenzyme Q10 (CoQ10) on the body weight and spleen and thymus indexes. **(A)** Body weight monitoring. **(B)** Spleen and thymus indexes analysis. The data represent means ± SD (*n* = 10). n.s., not significant; compared to the control group.

We conducted *in vivo* assays to investigate the effects of various concentrations of CoQ10 on the immune function using Kunming mice. Firstly, the body weight changes were monitored during the whole animal experiment. After treated for 4 weeks, no significantly difference the average body weight between the control group and the CoQ10 groups (Figure 1A, *P* > 0.05). For evaluating the effects of CoQ10 on the health of mice, various tissue indices were analyzed, mainly because the tissue index can reflect the potential effects of drugs on various organs, thus helping to assess the safety, toxicity and effectiveness. The spleen and thymus have played vital roles in the immune system, each with its own unique function to maintain the body’s immune balance and health (27, 28). Thus, the spleen and thymus index were calculated after the mice were sacrificed. As shown in Figure 1B, the values of both spleen and thymus index between control group and CoQ10 groups did not show any significant differences, indicating that the oral administration of CoQ10 showed negligible impact on the mouse body weight and tissues index. The absence of notable alterations in body weight and organ indices indicates that the immunomodulatory effects of CoQ10 are not a consequence of systemic metabolic changes, but rather arise from specific cellular mechanisms. This observation aligns with the effects of conjugated linoleic acids, which enhance immune function without influencing body composition (29).

### 3.2 Effect of CoQ10 on cellular immune response in mice

**Figure 2:**
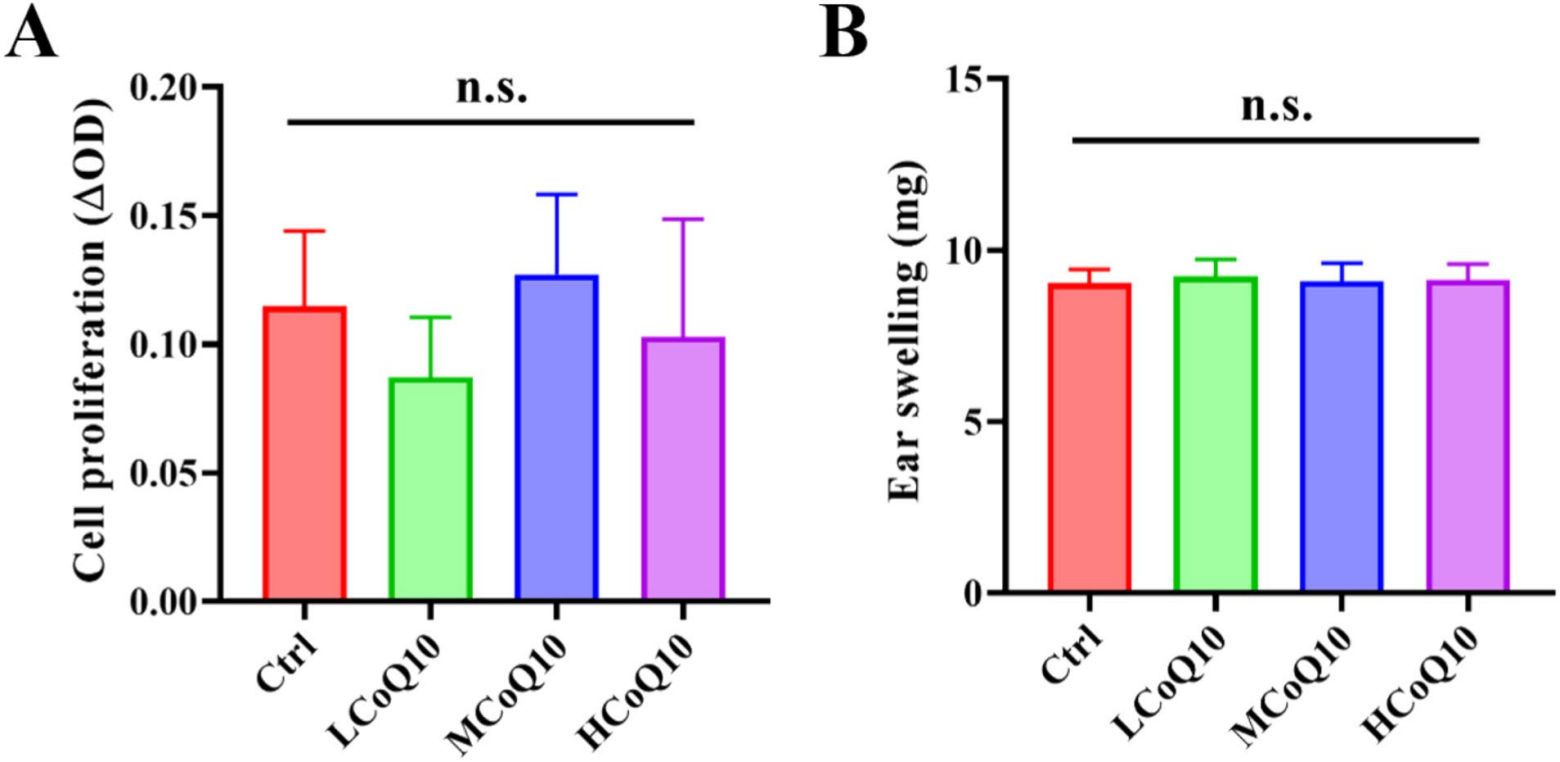
Effect of Coenzyme Q10 (CoQ10) on the mouse cellular immune function. **(A)** The cell proliferation ability evaluation after the mice were treated by CoQ10 using splenic lymphocyte transformation assay. **(B)** Ear swelling test after the mice were treated by CoQ10 using DTH response assay. The data represent means ± SD (*n* = 10). n.s., not significant; compared to the control group.

The utilization of lymphocyte proliferation assays has become a prevalent practice for assessing cell-mediated immune responses, offering a comprehensive evaluation of the body’s ability to mount an immune defense mediated by lymphocytes (30). In this study, the ConA-induced mouse splenic lymphocyte transformation assay was performed to evaluate the effect of CoQ10 on the cellular immune function. As shown in Figure 2A, it is evident that the oral administration of varying doses of CoQ10 to mice over a 30-day period did not elicit a notable alteration in the proliferation capacity of lymphocytes, as compared to the untreated control group (*p* > 0.05). This observation underscores the lack of a significant influence of CoQ10 on the ability of mouse lymphocytes to undergo transformation, thereby indicating no apparent stimulatory or inhibitory effects on this vital cellular function.

The extensively utilized DTH experimental paradigm serves as a versatile tool for evaluating the immunomodulatory and anti-inflammatory potential of a diverse array of novel compounds and materials, specifically with regard to their influence on the intricate T-cell-mediated immune response (31). So, the DTH response assay was carried out to reflect the impact of CoQ10 on the mouse cellular immune response. As shown in Figure 2B, after 30 days administration of CoQ10, average ear swelling value of all CoQ10 groups was similar to the control, showing no significant difference (*p* > 0.05), and indicating that CoQ10 did not show any impact on the DTH response ability of mice. It is worth noting that the cellular immune system is composed of a highly complex and intricate network of cells and molecules that interact in a precise and dynamic manner. Moreover, it is important to consider the potential for inter-individual variability in the immune responses of mice. So, in light of these considerations, it is premature to conclude that CoQ10 has no impact on cellular immune function in mice. Rather, our results suggest that under the specific experimental conditions employed in this study, CoQ10 did not elicit a notable effect on the proliferation capacity of lymphocytes or on the delayed-type hypersensitivity response.

### 3.3 Effect of CoQ10 on the mouse humoral immune function

**Figure 3:**
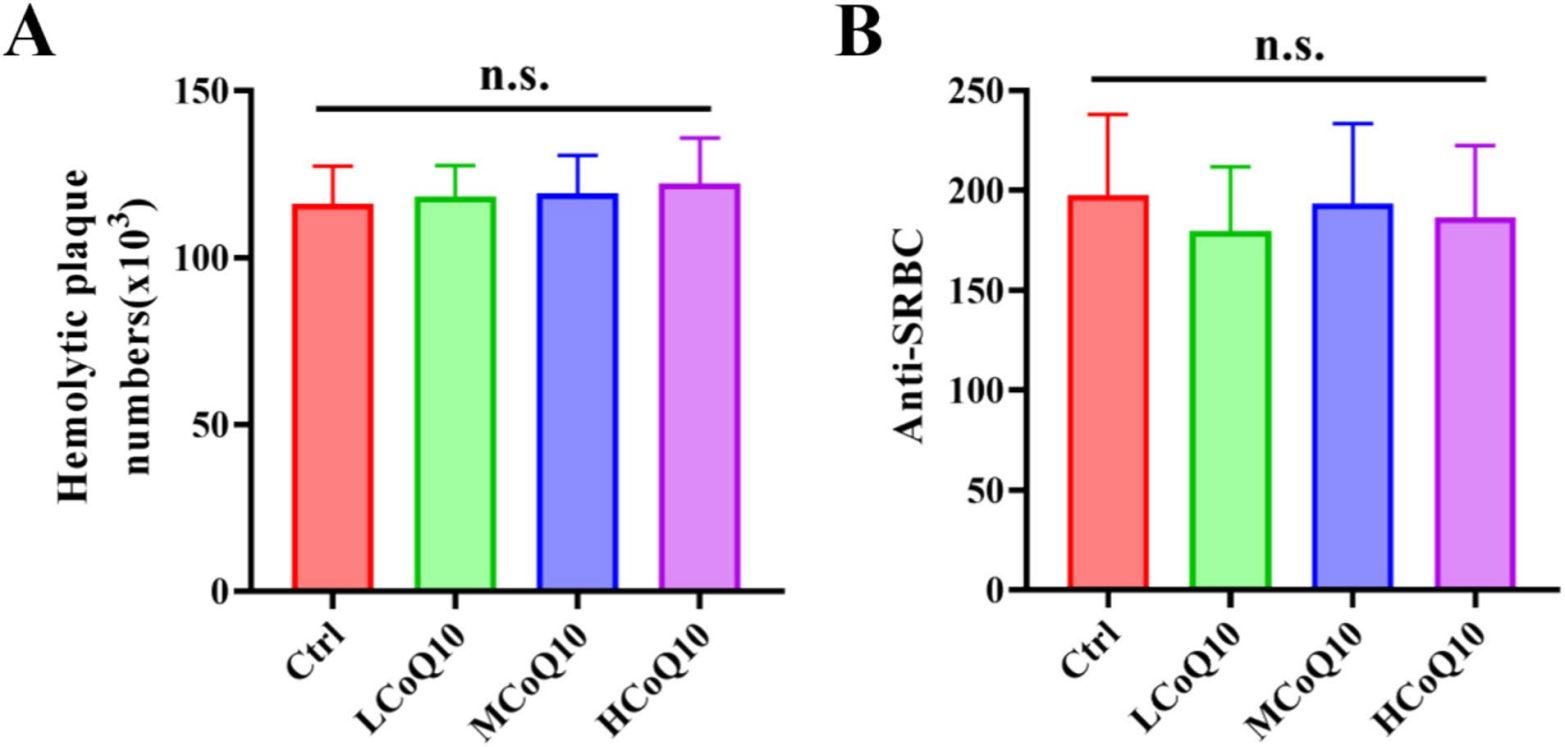
Effect of Coenzyme Q10 (CoQ10) on the mouse humoral immune function. **(A)** The number of hemolytic plaques, and **(B)** Anti-SRBC values after the mice were treated by CoQ10 for 30 days. The data represent means ± SD (*n* = 10). n.s., not significant; compared to the control group.

Apart from the cellular immune function, we also probed the effect of CoQ10 on the mouse humoral immune using hemolytic antibody plaques formation test. As shown in Figure 3A, hemolytic plaques for the control cohort attains a value of 1.16 × 10^5^, while after treated by different concentrations of CoQ10, the hemolytic plaques were 1.16 × 10^5^, 1.19 × 10^5^ and 2.22 × 10^5^, respectively. The analysis revealed no statistically significant differences (*p* > 0.05) in the count of antibody-producing cells between the control cohort and those administered with CoQ10. In essence, the administration of CoQ10 failed to elicit a noteworthy effect on the quantity of these cells in the mice, underscoring the absence of a pronounced impact on the immune response under investigation.

To further elucidate the influence of CoQ10 on the humoral immune response in mice, we assessed the serum hemolysin levels employing the anti-SRBC assay. Figure 3B presented the anti-SRBC values after the mice were treated by CoQ10 for 30 days, in which, the measured average values among the CoQ10-treated groups exhibited no statistically significant deviation from those of the control group (*p* > 0.05). This finding implies that the administration of CoQ10 did not elicit any discernible effect on the quantity of antibody-producing cells in the experimental mice. The above results showed that the CoQ10 treatment did not show any obvious impact on the mouse humoral immune function.

It is important to acknowledge that humoral immunity encompasses a complex interplay of multiple factors, including B-cell activation, antibody production, and immune complex formation, among others. Therefore, the lack of a notable effect on antibody-producing cells as observed in our study does not necessarily preclude the possibility of CoQ10 modulating other aspects of humoral immunity. Furthermore, it is crucial to consider the experimental design and conditions that may have influenced our results. For instance, the dosage and duration of CoQ10 administration, as well as the specific strain and immunological status of the mice used, could potentially impact the observed outcomes. Therefore, future studies examining the effects of CoQ10 on humoral immunity may benefit from exploring a broader range of experimental conditions and parameters.

### 3.4 Effect of CoQ10 on the phagocytic function of mononuclear macrophage

**Figure 4:**
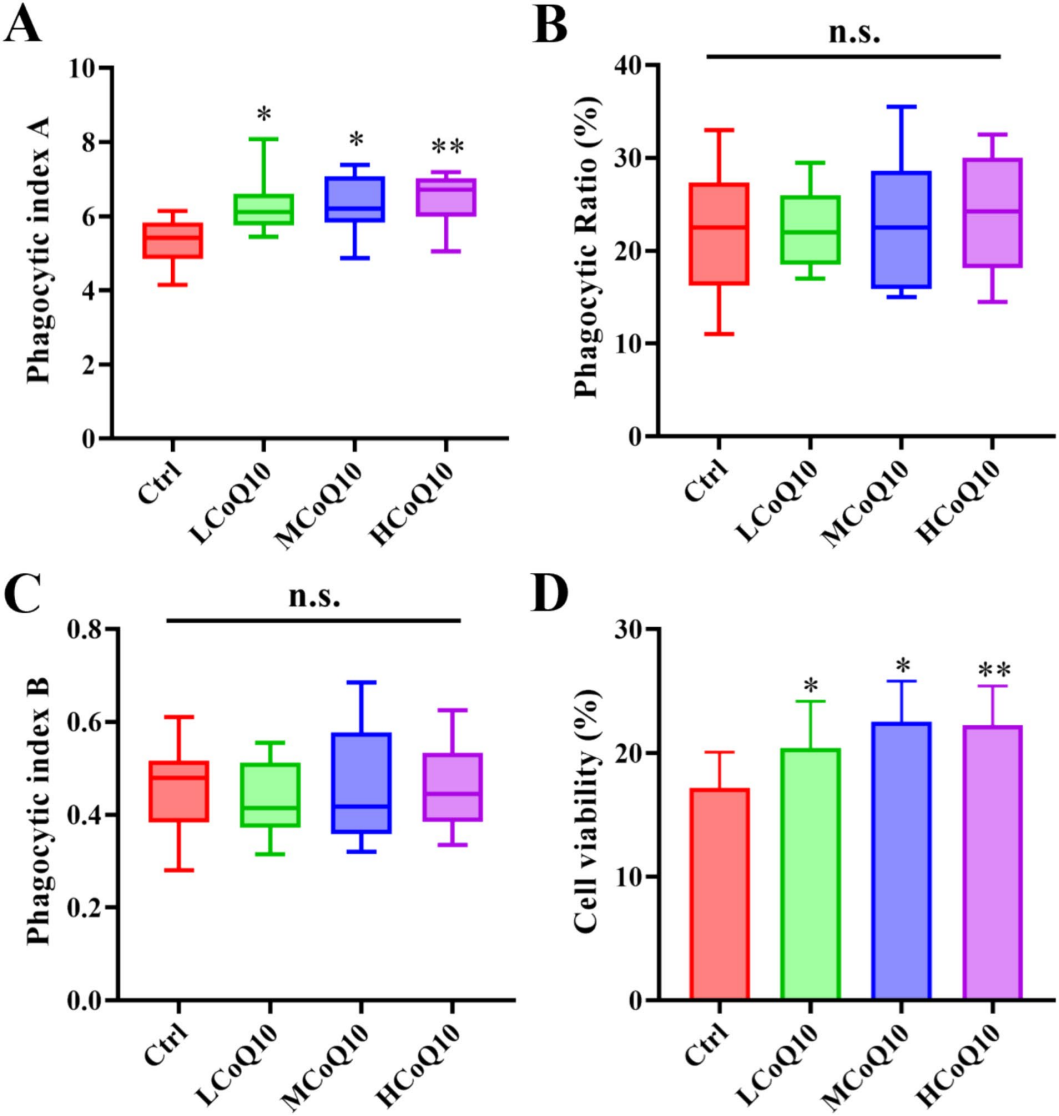
Effect of Coenzyme Q10 (CoQ10) on the mouse phagocytic function of mononuclear macrophage. **(A)** Carbon clearance test. **(B)** Phagocytic ratio, and **(C)** phagocytic index B of peritoneal macrophage englobing chicken erythrocytes. **(D)** NK cellar viability after the mice were treated by CoQ10 for 30 days. The data represent means ± SD (*n* = 10). *, *p* < 0.05; **, *p* < 0.05; n.s., not significant; compared to the control group.

The carbon clearance assay is a pivotal technique for investigating immune reactions and the operational dynamics of macrophages by monitoring their clearance efficiency of administered carbon particles, which offers a comprehensive assessment of the immune system’s functionality, with a particular focus on the reticuloendothelial system (32, 33). Furthermore, it quantifies macrophage capacity to eliminate carbon particles from the circulatory system, yielding valuable insights into potential immunomodulatory impacts. And the carbon clearance assay is also used to test non-specific immune response, and clinical studies and diagnostics (32, 33). Therefore, we probed the effect of CoQ10 on carbon clearance ability of mouse monocytes-macrophages. The phagocytic index A was calculated, and the outcomes revealed a notable elevation in the phagocytic index A across all groups treated with CoQ10, in stark contrast to the control group (Figure 4A, *P* < 0.05), which implied that administration of CoQ10 remarkably enhanced the carbon clearance ability of monocytes-macrophages. However, the CoQ10 did not present any obvious influence on the phagocytic ability of peritoneal macrophage englobing chicken erythrocytes. The data of both the phagocytic ratio and phagocytic index B of the CoQ10 treatment groups were similar to the control group, which showed no significant difference among different groups (Figures 4B,C, *P* > 0.05). The significant elevation in phagocytic index A, indicating enhanced carbon clearance ability of monocytes-macrophages in response to CoQ10 treatment, suggests that CoQ10 may possess immunostimulatory properties that specifically target this phagocytic cell type. Given that carbon clearance assays are commonly used to assess the functional activity of phagocytes, the results here suggest that CoQ10 may enhance the ability of monocytes-macrophages to engulf and remove foreign particles, such as carbon particles, from the circulation. This finding aligns with previous studies that have reported the antioxidant and anti-inflammatory properties of CoQ10, which could potentially contribute to improved phagocytic function (34). However, the lack of effect on the phagocytic ability of peritoneal macrophages engulfing chicken erythrocytes is intriguing, suggesting that the immunostimulatory effects of CoQ10 may be cell-type specific or dependent on the type of phagocytic target. Another possibility is that the phagocytic assay using chicken erythrocytes may not be sensitive enough to detect subtle changes in phagocytic function induced by CoQ10.

The NK cells are known for their rapid response to infected or transformed cells and play a crucial role in innate immunity. So, the viability of NK cells was also examined after the mice treated by CoQ10 for 30 days. As shown in Figure 4D, the viability of cells in all three experimental groups treated with CoQ10 exhibited a statistically significant elevation compared to the untreated control group (*p* < 0.05). This observation underscores the potential of CoQ10 administration to effectively bolster the functional capacity of NK cells. From the above results, it was evident that although administration of CoQ10 showed negligible impact on the function of phagocytic ability on chicken erythrocytes, the activities of carbon clearance and phagocytic NK cells were significantly enhanced, which implying that the function of mouse monocyte–macrophage phagocytosis was enhanced to a certain extent after administrated of CoQ10 administration. By promoting NK cell viability, CoQ10 may contribute to the overall effectiveness of the immune system in combating pathogens and maintaining health. Moreover, the fact that CoQ10 had a negligible impact on the phagocytic ability of peritoneal macrophages towards chicken erythrocytes, but significantly enhanced carbon clearance and phagocytic activity in monocytes-macrophages, suggests a level of specificity in its immunomodulatory effects. This specificity may be related to the different roles and functional specializations of these cell types within the immune system. It is also noted that the effects of CoQ10 on immune function may be mediated through multiple mechanisms (35). As an essential component of the electron transport chain in mitochondria, CoQ10 plays a critical role in energy production and oxidative phosphorylation. Its antioxidant properties may also help to protect cells from oxidative stress and damage caused by ROS. Both of these mechanisms could contribute to the observed enhancement of NK cell viability and monocyte–macrophage phagocytosis. Although the exact mechanisms underlying these effects remain to be fully elucidated, the findings suggest that CoQ10 may be a useful immunomodulatory agent with broad implications for the prevention and treatment of immune-related disorders. Further research is needed to explore the full extent of CoQ10’s immunomodulatory effects and to determine the optimal dosing and administration strategies for maximizing its therapeutic potential.

### 3.5 Effect of CoQ10 on the mouse gut microbiota

**Figure 5:**
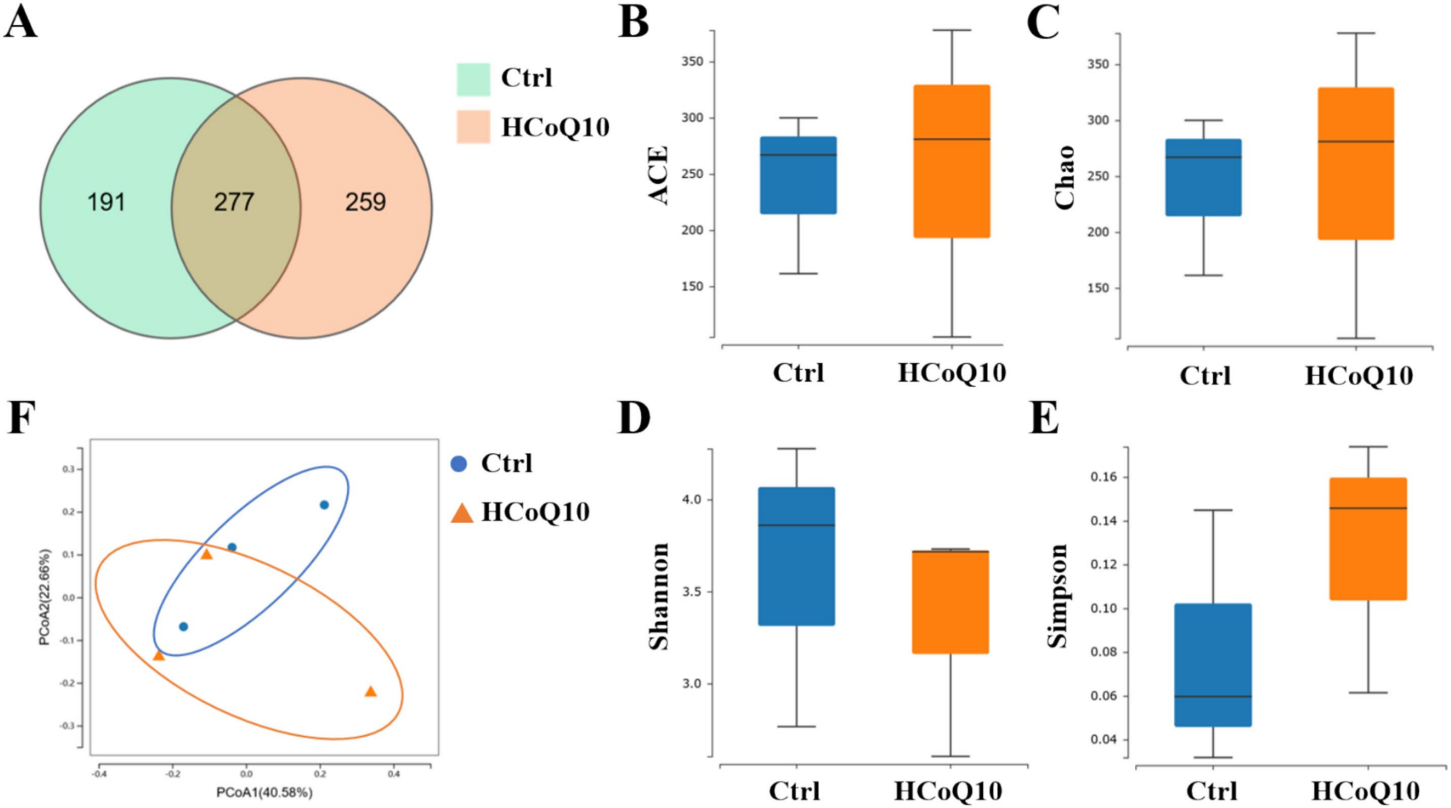
Effect of Coenzyme Q10 (CoQ10) on the richness and diversity of gut microbial communities in mice. **(A)** Venn diagram. The *α*-diversity analysis of **(B)** ACE, **(C)** Chao, **(D)** Shannon, and **(E)** Simpson. **(F)** PCoA plot analysis.

Recent advancements in research have conclusively demonstrated the pivotal roles of gut microbiota homeostasis in modulating the body’s immune system functions (21, 36). Preliminary physiological and biochemical analyses have indicated that the lower and middle doses of CoQ10 showed similar effects to the higher dose CoQ10 on the mononuclear macrophage and NK cell activity, but the influence of lower and middle doses might be more subtle or require more sensitive detection methods. Hence, we investigated the alterations of gut microbiota-associated indices in mice administered with high-dose CoQ10 (HCoQ10) utilizing 16S rDNA gene sequencing. Notably, 277 operational taxonomic units (OTUs) were shared between the control and HCoQ10 cohorts, whereas 191 OTUs were exclusive to the control group and 259 OTUs were exclusive to the HCoQ10 group, indicating that administration of CoQ10 obviously increased microbial flora species compared to the control group (Figure 5A). The *α*-diversity analysis, depicted in Figures 5B–E, failed to discern any notable variations in the richness and diversity patterns of microbial communities when comparing the control group with HCoQ10-treated group, as evidenced by the comparable α-diversity indices of ACE, Chao, Shannon, and Simpson. Furthermore, to assess the compositional distinctiveness and potential variations in α-diversity, we applied weighted UniFrac principal component analysis. Intriguingly, despite some overlapping species, the PCoA plot vividly segregated the HCoQ10 group from the control group, highlighting a distinct clustering pattern (Figure 5F). These findings underscore the capacity of CoQ10 administration to reshape the intestinal microbial structures in mice.

**Figure 6:**
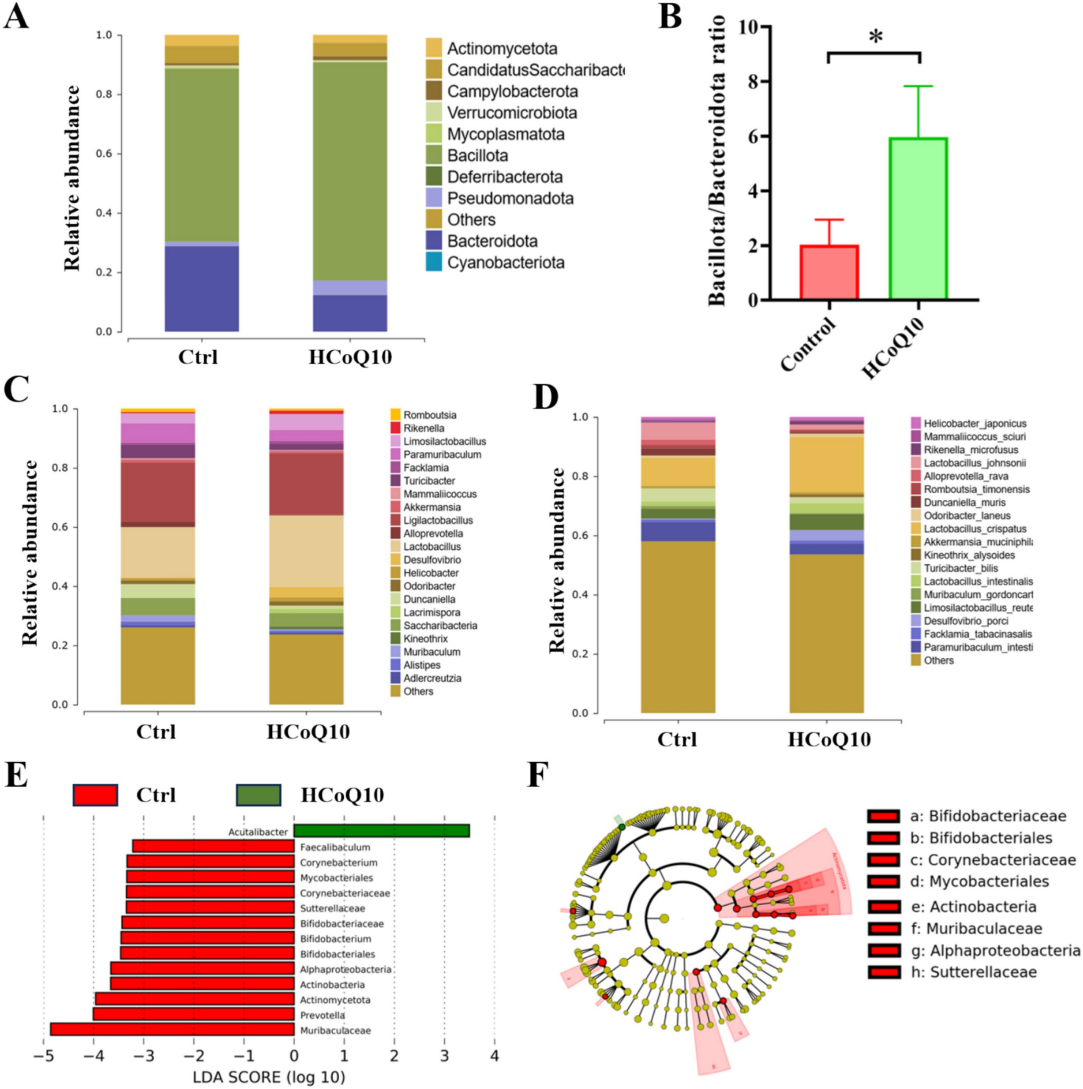
Effect of Coenzyme Q10 (CoQ10) on the structure and composition of gut microbial communities in mice. **(A)** Relative abundance of bacteria at phylum level. **(B)** The ratio of *Bacillota*/*Bacteroidota*. Relative abundance of bacteria at **(C)** genus, and **(D)** species levels. **(E)** LDA analysis histogram, and **(F)** LEfSe Analysis Cluster comparison of gut microbiota between the control and HCoQ10 groups. *, *p* < 0.05; compared to the control group.

Subsequently, we examined the nuanced disparities in intestinal flora composition across the mice within each group at varying hierarchical levels. At the phylum level, a prevalent pattern emerged, with the intestinal microbiota of mice in all groups predominantly consisting of *Bacillota* and *Bacteroidota*, and showcasing a conservation amidst diversity (Figure 6A). The relative abundance of *Bacillota* in HCoQ10 was increased, and *Bacteroidota* abundance was decreased than the control group, resulting an enhancement of *Bacillota*/*Bacteroidota* ratio in CoQ10 treatment group (Figure 6B). The relative abundances of *Rikenella, Lactobacillus*, *Limosilactobacillus*, *Lacrimispora* in HCoQ10 group were increased, but the relative abundances of *Paramuribaculum*, *Duncaniella*, *Turicibacter*, *Muribaculum* were decreased compared to the control group at the genus level (Figure 6C). Further analysis at species level showed that the relative abundances of *Lactobacillus crispatus*, *Lactobacillus intestinalis*, *Limosilactobacillus reuteri* were enriched, and relative abundances of *Paramuribaculum intestinale*, *Turicibacter bilis* were decreased in the HCoQ10-treated group compared with the control cohort (Figure 6D). The elevated abundance of beneficial microbial populations might contribute to the improvement on immunity in mice, for example, as reported that the surface-layer protein secreted by *Limosilactobacillus crispatus* presented an immunoregulation effect by autophagy involved the PI3K/AKT/mTOR signal pathway (37). *Lactobacillus reuteri* possesses the capability to excrete a unique, non-proteinaceous, broad-spectrum antibacterial agent denominated as roxithromycin, and this antimicrobial substance effectively inhibits the proliferation of detrimental bacterial strains, maintains the intestinal mucosal barrier integrity, and concurrently alleviates inflammatory responses (38). These findings demonstrated that CoQ10 has the capacity to reshape the architecture and microbial profile of the intestinal microbiota in mice, specifically by fostering the enrichment of advantageous microbial strains, and reducing the harmful strains, therefore achieving the effect of immunoregulation function.

Furthermore, we employed the LEfSe, a specialized LDA-driven approach integrating nonparametric Kruskal–Wallis and Wilcoxon tests, to identify pivotal biomarkers distinguishing between the control and HCoQ10 groups. Figures 6E,F illustrated both the histogram representation of LDA scores (scores ≥4) and the evolutionary clustering analysis chart showcasing the taxonomic profiles of the crucial microbial flora. Interestingly, *Acutalibacter* was the main biomarker in the HCoQ10 group, and *Muribaculaceae*, *Bifidobacteriaceae*, *Prevotella*, etc. were biomarkers in the control group (Figures 6E,F). These data further showed that administration of CoQ10 altered the gut microbiota compositions in mice. Furthermore, Pearson Correlation Analysis was conducted between representative bacteria occupancy and phagocytic index or cell viability, and the results showed that CoQ10 administration was strikingly positively associated with the enhancement of carbon clearance and NK cell activity, while a significant positive correlation with the abundance of *Rikenella* and a notable negative correlation with that of *Muribaculum* was also observed.

Our present findings, despite being centered on the 30-day CoQ10 intervention period, offer invaluable insights into the impact of CoQ10 supplementation on gut microbiota. Recognizing the potential value of baseline microbiota data, we suggest that future research endeavors should incorporate a comparative analysis of the pre-and post-intervention microbial profiles within individual mouse. This approach would enhance our understanding of the dynamic microbial shifts in response to interventions, thereby contributing to the development of more targeted and effective therapeutic strategies. In addition, our current work was aimed to establish a fundamental understanding of the immunomodulatory effects of CoQ10 through its interaction with gut microbiota, using a dose that showed robust effects. Therefore, our focus on the higher concentration dose of CoQ10 was a strategic choice to ensure a robust and interpretable outcome. Moreover, as seen in meta-analyses of royal jelly (2) and conjugated linoleic acids (29), the clinical translation of natural compounds requires careful consideration of dosage, duration, and target populations. Future studies should explore dose–response relationships and long-term safety profiles to optimize CoQ10’s therapeutic potential.

## 4 Conclusion

In conclusion, our study has systematically evaluated the effects of CoQ10 on the mouse immune system. We found that although the CoQ10 showed negligible influence on the body weight and tissue indices of mice, enhanced mouse immunity was observed as shown by elevated carbon clearance ability and NK cellar viability. Moreover, 16S rRNA gene sequencing revealed that CoQ10 administration altered the structure and compositional landscape of the gut microbiota in mice, as reflected in increased abundances of *Lactobacillus*, *Limosilactobacillus*, and decrease abundance of *Paramuribaculum*.

## Data Availability

The original contributions presented in the study are included in the article/supplementary material, further inquiries can be directed to the corresponding authors.
